# The Incretin Mirage: Epigenetic Memory as a Potential Driver of Post-Treatment Weight Regain^☆^

**DOI:** 10.1016/j.advnut.2026.100737

**Published:** 2026-09-17

**Authors:** Ruslan Kubant, G Harvey Anderson

**Affiliations:** 1 Department of Nutritional Sciences, University of Toronto, Toronto, ON, Canada; 2 Department of Physiology, University of Toronto, Toronto, ON, Canada

**Keywords:** obesity, GLP-1/GIP receptor agonists, weight regain, translational medicine, adipose tissue memory, Epigenetic Detent, Incretin Mirage

## Abstract

Although incretin receptor agonists deliver profound weight loss, clinical data reveal a consistent challenge: treatment discontinuation routinely triggers rapid weight regain. We define this phenomenon as the “Incretin Mirage”—a state where pharmacologically induced weight reduction temporarily conceals the persistent metabolic and neuroendocrine adaptations established during chronic obesity. Despite a lower body mass, key drivers of the disease, including adipocyte dysfunction and altered appetite signaling, retain a molecular imprint of obesity driven by persistent epigenetic marks. To explain why the body aggressively resists sustained weight loss after treatment cessation, we introduce the “Epigenetic Detent.” This novel conceptual mechanism links adipocyte epigenetic memory with neuroendocrine plasticity to preserve a high-adiposity set-point. Consequently, the moment pharmacological suppression is lifted, this persistently defended set-point serves as a potential driver of weight regain. Achieving a durable therapeutic response therefore requires moving beyond transient appetite suppression. We advocate for a coordinated, multitiered strategy that couples lifestyle and environmental restructuring with emerging epigenetic therapies aimed at erasing these cellular marks and achieving a complete cellular metabolic reset.

**Statement of significance**

Existing literature incompletely explains why weight loss from incretin therapies routinely reverses on drug cessation, in part because current clinical endpoints prioritize the scale over the cell. This perspective addresses this gap by proposing a novel translational model that links adipocyte epigenetic memory to post-treatment weight regain, alongside a needle-biopsy profiling approach to track a complete cellular metabolic reset.

## Introduction

The emergence of incretin-based therapies has transformed obesity management, underscoring that obesity is an actively defended homeostatic state. Driven by biological hysteresis—the path-dependent regulation by which a system’s response depends on its prior trajectory rather than its current input alone—the physiological trajectory of weight reduction is not a simple inverse of the path to weight gain. This hysteretic regulation suggests that the body preserves a highly persistent memory of its peak adiposity, a defense mediated through neuroendocrine pathways and sustained epigenetic marks. These involve stable changes in chromatin accessibility and DNA methylation that actively maintain a high-adiposity set-point [[1], [2], [3]].

This defense system operates through evolutionarily conserved biological feedback loops. In humans, these circuits trigger powerful counter-regulatory responses whenever body fat stores are rapidly depleted, driving compensatory hunger, lower energy expenditure, and the preferential recovery of adipose tissue over lean mass [4,5]. This homeostatic drive ensures that weight reduction remains a metabolically defended state, governed by complex interorgan communication across the gut–brain axis.

Although incretin receptor agonists achieve substantial weight reduction, rapid weight regain typically follows medication discontinuation. This relapse pattern demonstrates that pharmacologic appetite suppression masks, rather than erases, the underlying biology of chronic obesity. We propose the “Incretin Mirage” to describe this clinical state. Importantly, this descriptor does not imply that the phenotypic weight reduction is an illusion; the loss of adipose mass on the scale is a profound, clinically verified reality. Rather, the “Mirage” conceptualizes the stark physiological dissociation that occurs during treatment, where a visibly leaner body temporarily conceals an uncorrected cellular state that remains molecularly programmed for the prior obese trajectory. Although this biological masking may apply to pharmacologically induced weight loss in general—including next-generation, nonincretin-based multiagonists currently in development—we center this conceptual model explicitly on incretin-based therapies, which have brought the challenge of post-treatment weight regain to the forefront of the public health crisis. To explain why this rebound occurs, we introduce the “Epigenetic Detent”—a mechanism through which developmental programming, cell-autonomous adipocyte persistence, and central neuroendocrine adaptations collectively anchor the body to a high-adiposity set-point. Modern obesogenic exposures continuously exploit this persistent molecular state, driving a stark dissociation between phenotypic weight loss and a complete cellular metabolic reset.

## Mechanisms of the Epigenetic Detent

The Epigenetic Detent operates at the intersection of evolutionary physiology and modern environmental pressures. Rather than acting as a static byproduct of prior weight gain, the Detent functions as an active biological defense—a discrete, state-locking molecular mechanism that requires sustained counter-regulatory force to overcome (Figure 1).

**FIGURE 1:**
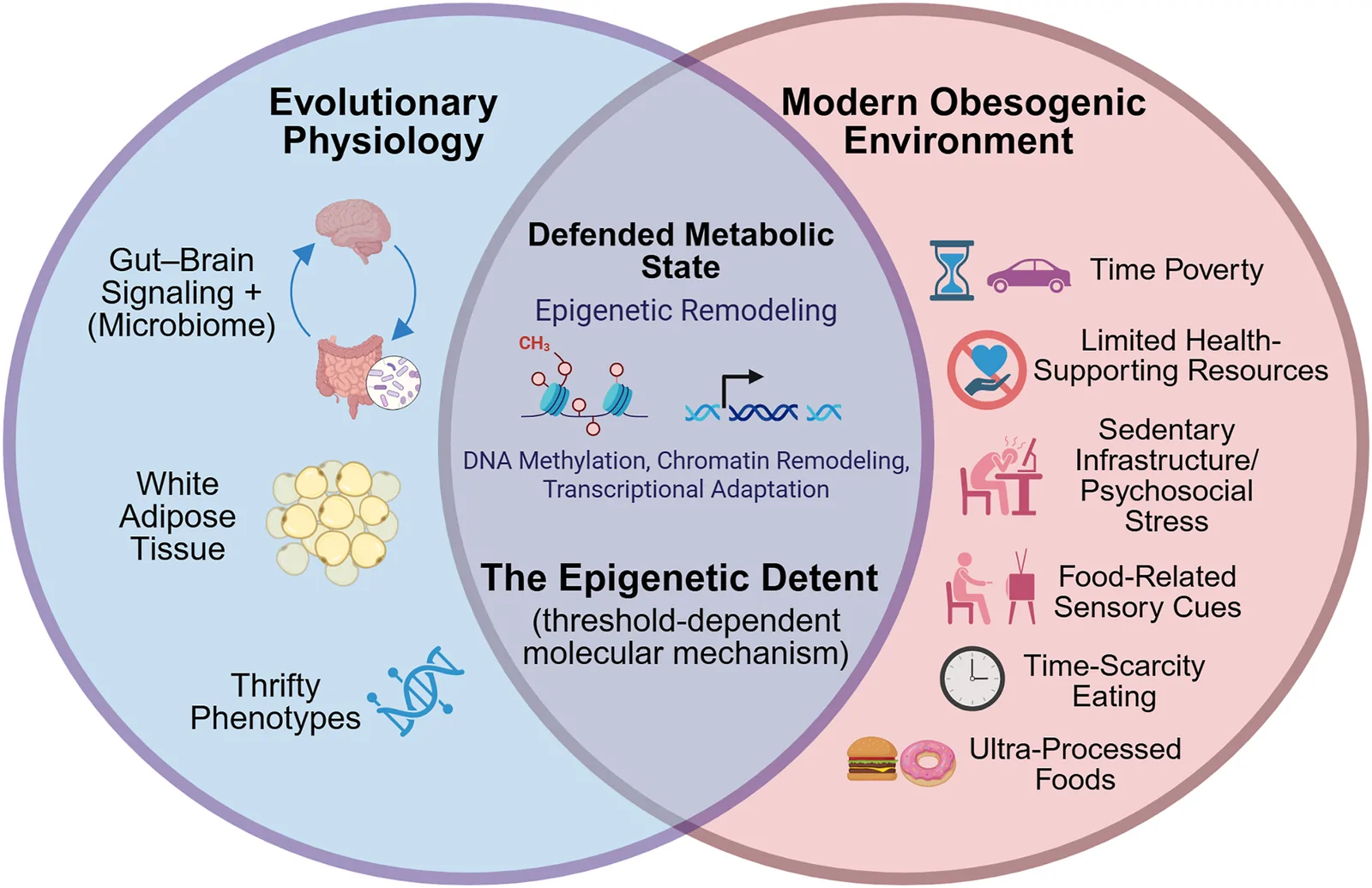
The Epigenetic Detent. This conceptual schematic maps how modern environmental exposures interact with evolutionarily conserved biological pathways to perpetuate obesity. The left panel (evolutionary physiology) outlines survival networks that prioritize energy conservation through gut-brain-microbiome signaling, the maintenance of white adipose tissue, and thrifty phenotypes. Conversely, the right panel (Modern Obesogenic Environment) introduces distinct modern environmental pressures, including time poverty and time-scarcity eating patterns, limited health-supporting resources, sedentary infrastructure, psychosocial stress, food-related sensory cues, and ubiquity of ultra-processed foods. The central intersection of these domains drives the stable molecular adaptations that constitute the Epigenetic Detent, maintaining the organism in a persistently defended metabolic state. Here, the Detent operates as a threshold-dependent molecular mechanism, maintaining a high-adiposity set-point through altered DNA methylation, chromatin remodeling, and transcriptional adaptation. Created in BioRender. R. Kubant (2026). https://BioRender.com/pbmw3ma.

This mechanism maintains a high-adiposity set-point against standard therapeutic interventions, yet remains inherently reversible, capable of disengagement once targeted counter-regulatory forces cross a physiological threshold. Although central neuroendocrine networks dictate satiety, human white adipose tissue independently preserves inflammatory and lipogenic transcriptional signatures despite weight loss, creating a persistent resistance to sustained adiposity reduction [3].

Developmental exposures occurring during critical early-life windows induce highly persistent biological programming, although these shifts remain bounded by developmental plasticity and may permit phenotypic switching between metabolic set-points [6]. Epigenetic processes, such as DNA methylation, link transient environmental stressors to long-term shifts in gene expression. In Gambian infant cohorts, seasonal maternal nutritional fluctuations program the DNA methylation baseline of the offspring’s peripheral pro-opiomelanocortin (*POMC*) locus, providing a blood-accessible surrogate of central appetite circuits [7]. These modifications act as stable epigenetic marks that sustain the Epigenetic Detent long after the primary stimulus is removed—a phenomenon mirrored in *Agouti* mouse models where maternal gestation diets dictate offspring phenotypes through the same epigenetic mechanism [8]. Similarly, the Pune Maternal Nutrition Study demonstrates that maternal micronutrient imbalances (high folate, low vitamin B-12) drive elevated adiposity and insulin resistance in children by age 6 y [9], aligning with preclinical work showing that gestational folic acid supplementation alone primes rat offspring for adult hyperphagia and obesity [10].

Importantly, adult weight management operates under entirely different biological constraints than these early-life windows. Although early-life exposures establish a highly stable metabolic baseline, adult dietary or lifestyle changes alter only a few temporary markers rather than permanently resetting the body’s underlying cellular memory. Because long-term epigenetic cohorts tracking patients after the withdrawal of incretin therapies do not yet exist, human bariatric surgery and multi-intervention datasets provide a compelling proof-of-concept for adult adipocyte memory. Even though the clinical settings differ, these data serve as a valuable mechanistic model for understanding how tissue-level epigenetic memory persists. For instance, bariatric surgery alters 3,601 cytosine-phosphate-guanine (CpG) sites in subcutaneous adipose tissue [11], and a 6-month exercise regimen remodels 17,975 adipose CpG sites [12] alongside *ADIPOR1* and *TRIB1* skeletal muscle promoters [13]. Although bariatric surgery and exercise alter thousands of loci, these modifications represent transient regulatory fluctuations in tissue plasticity rather than an erasure of the core epigenetic marks that sustain the underlying Epigenetic Detent.

Furthermore, lifestyle-induced remodeling remains gradual and modest in magnitude [14]. Data indicate that while thousands of loci fluctuate during weight loss, a core subset of obesity-associated epigenetic signatures is preserved [3], sustaining weight-regain susceptibility. Although environmental factors generate temporary histone alterations, they often fail to drive stable DNA demethylation via ten-eleven translocation (TET) enzymes [15], leaving baseline metabolic programming intact. Functionally, these epigenetic modifications govern lipogenesis and adipocyte differentiation pathways, as reinforced by animal models and human epigenome-wide association studies (EWAS). Key targets include *Srebf1*-driven lipogenic genes (*Acaca* and *Fasn*) alongside *Pparg*, the master regulator of lipid uptake [16], matching human EWAS data linking BMI to specific methylation changes [17,18]. Adipocyte persistence further exacerbates this susceptibility; whereas cell volume shrinks during weight loss, total adipocyte number remains highly stable [19], preserving lipid-storage capacity despite substantial reductions in body weight. Single-nucleus mapping reveals the molecular basis of this tissue-level retention, demonstrating that prior obesity leaves a persistent epigenetic mark on fat cells [20]. Although parallel mouse models show that this memory is driven by a long-lasting gain in repressive histone modifications such as H3K27me3, human adipocytes mirror this retention through the enduring downregulation of key metabolic genes, including *IGF1*, *LPIN1*, and *DUSP1* [20]. Whether these epigenetic alterations function as primary causal drivers, downstream consequences, or components of a broader adaptive complex remains an active area of investigation [17,18,21].

## Environmental Reinforcement and the Path to Remission

Modern environmental pressures—including time poverty, psychosocial stress, sedentary infrastructure, and the ubiquity of ultra-processed foods—interact to disrupt physiological appetite regulation (Figure 1). When these chronic socioenvironmental stressors exploit evolutionarily conserved pathways favoring energy conservation, they produce a sustained mismatch between caloric intake and metabolic demand. This continuous interaction with pre-existing biological networks actively reinforces the Detent through coordinated neuroendocrine and epigenetic mechanisms [22]. As demonstrated by the metabolic ward trials from Hall et al. [23], ultra-processed foods drive an increased rate of consumption that delays gut–brain satiety signaling, resulting in an involuntary caloric surplus of ∼500 kcal/d. This decoupling is further exacerbated by a dysbiotic gut microbiome, driven by the consistent loss of microbial diversity in contemporary dietary environments [24,25]. This environmentally reinforced dysregulation of appetitive behavior is the behavioral expression of the underlying Epigenetic Detent. Driven by altered methylation at obesity-associated loci such as *SH2B1*, modern obesogenic exposures can continuously trigger the hunger responses dictated by this molecular mechanism [17].

Furthermore, persistent environmental pressures sustain the biological architecture favoring positive energy balance. Overcoming the post-treatment homeostatic rebound requires moving beyond continuous, single-pathway appetite suppression toward multitargeted strategies engineered to disengage the Epigenetic Detent. As conceptualized in our model, this requires a synchronized, dual-pronged approach combining broad socioenvironmental restructuring with targeted molecular interventions during the active antiobesity medication window, before treatment withdrawal. During this period, sustained caloric deficit and reduced insulin signaling create the permissive metabolic state in which epigenetic remodeling is most likely to take hold.

First, structural modifications of the immediate food environment are necessary to reduce the artificial sensory cues that exploit the path-dependent epigenetic state established by the Detent. Replacing ultra-processed foods with accessible, nutrient-dense alternatives blunts the hyperinsulinemic and inflammatory stimuli that drive peripheral lipogenic pathways. To maximize efficacy, these dietary modifications should be coordinated with microbiome-directed therapies. Restoring gut microbial diversity through nutritional inputs helps repair disrupted gut–brain satiety signaling, reducing the external drivers that sustain the Detent.

Second, this nutritional restructuring must be accompanied by specific molecular and cellular interventions to erase entrenched adipocyte epigenetic marks. Rather than relying on passive weight maintenance, clinicians can accelerate the disengagement of the Epigenetic Detent through several emerging translational pathways. These include pharmacological epigenetic modulators, such as small-molecule inhibitors of specific histone deacetylases or DNA methyltransferases, to drive the erasure of the adipocyte epigenetic marks that weight loss alone does not correct [3]. Interventions that upregulate TET enzyme activity can further enhance active DNA demethylation and restore baseline promoter accessibility [15]. Translational strategies using experimental clustered regularly interspaced short palindromic repeats (CRISPR)/catalytically dead CRISPR-associated protein 9 (dCas9)-based epigenome editing could eventually be used to directly target and reset hypermethylated obesity-associated loci within adipose depots.

Finally, these molecular inputs must be integrated with staged lifestyle interventions, such as high-intensity resistance training to remodel skeletal muscle promoters, and time-restricted fasting to realign peripheral circadian clocks. By coordinating these environmental, microbial, and molecular inputs during antiobesity pharmacotherapy, clinicians could systematically lower the defended set-point, transitioning the organism away from a transient, drug-dependent state into a sustainable state of complete cellular metabolic reset.

## The Incretin Mirage and Post-Treatment Relapse

Although incretin receptor agonists drive profound weight loss by suppressing appetite and food-reward signaling, major extension trials show that rapid weight regain consistently follows drug discontinuation. This relapse pattern demonstrates that transient appetite suppression merely conceals, rather than corrects, the deep-seated biology of chronic obesity. The apparent metabolic reset vanishes once receptor occupancy ends, revealing that the underlying cellular defenses remain entirely unchanged (Figure 2).

**FIGURE 2:**
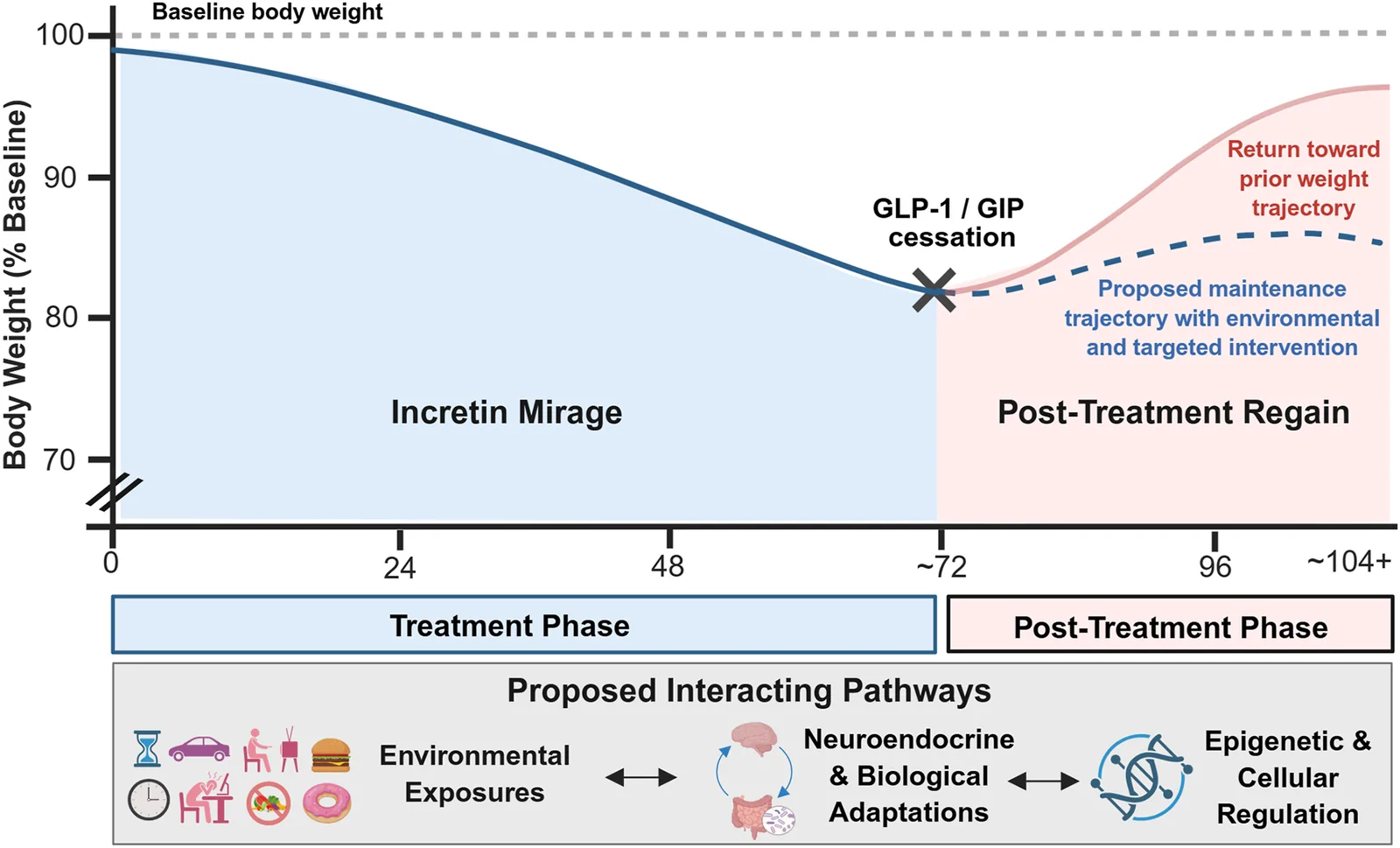
The Incretin Mirage and post-treatment relapse. Conceptual timeline of weight trajectories during and after incretin-based therapy. Data are synthesized from SURMOUNT-1 and SURMOUNT-4 (tirzepatide) trials and Semaglutide Treatment Effect in People with Obesity (STEP) 1 Extension trial. The upper panel depicts phenotypic trajectories across timelines (x-axis, weeks): the treatment phase establishes the Incretin Mirage, demonstrating substantial receptor agonist-driven weight loss while underlying molecular adaptations persist, whereas the post-treatment phase maps weight regain after glucagon-like peptide-1 (GLP-1)/glucose-dependent insulinotropic polypeptide (GIP) receptor agonist cessation. Removing pharmacological suppression allows the Epigenetic Detent to force body weight back toward its prior weight trajectory; the dashed line denotes the proposed maintenance trajectory with environmental and targeted intervention. The lower panel outlines the proposed interacting pathways, detailing the interplay between environmental exposures and neuroendocrine and biological adaptations to drive downstream epigenetic and cellular regulation via altered transcriptional and chromatin networks. Created in BioRender. R. Kubant (2026). https://BioRender.com/gpsykmt.

The treatment phase represents the active window of the Incretin Mirage. During this period, receptor agonists artificially blunt external sensory cues and suppress both homeostatic and hedonic appetite signaling. However, as observed at ∼72 weeks in the SURMOUNT-1 tirzepatide trial [26], this reduced body weight represents a temporary, drug-dependent deviation from a tightly defended high-adiposity set-point.

Conversely, the post-treatment phase marks the expected return of both homeostatic hunger drives and hedonic food cravings that follows drug withdrawal. Driven by biological hysteresis, body weight drops during active treatment, whereas peripheral and central defenses remain configured for the prior obese trajectory. On drug cessation, the Epigenetic Detent rapidly forces body weight back toward its high-adiposity set-point. Data from both SURMOUNT-4 [27] and the Semaglutide Treatment Effect in People with Obesity (STEP) 1 Extension trial [28] indicate that removing pharmacological suppression allows underlying biological adaptations to re-emerge. Because adipocyte epigenetic marks and neuroendocrine configurations remain uncorrected during treatment, the body resumes its defense of the prior metabolic set-point.

## Adolescent Development and Pediatric Considerations

Incretin-based therapies are now used in pediatric care. The American Academy of Pediatrics recommends offering incretin-based pharmacotherapies to patients aged ≥12 years alongside interventions for comorbidities, including dyslipidemia and hypertension [29]. Although these therapies provide vital short-term clinical benefits, deploying receptor agonists during adolescence overlaps with critical developmental windows of neuroendocrine maturation and epigenetic stabilization.

This sensitive developmental window makes the adolescent brain uniquely vulnerable to modern environmental pressures that exploit altered neurobehavioral circuits. For example, exposure to food advertising disrupts normal satiety responses in girls with a high BMI [30]. After exposure to food-related sensory cues, these girls eat more, allowing environmental inputs to systematically override both homeostatic and hedonic appetite signaling and drive overeating. A persistent gap in the field is our incomplete understanding of the neurocircuitry through which pediatric and adolescent populations regulate voluntary food intake [31]. Without clarifying these age-dependent physiological baselines, deploying incretin-based medications risks disrupting pathways that remain poorly mapped.

Although pharmacological treatment during these developmental periods attenuates body weight, it remains unknown whether early intervention prevents the establishment of the Epigenetic Detent or merely masks it during its formation. If underlying adipocyte epigenetic marks become fixed during adolescent development despite drug-induced weight loss, treatment cessation may trigger a pronounced homeostatic rebound during adulthood. This risks rendering early intervention a long-term pharmacological dependence rather than a durable, time-limited treatment. Mapping these tissue-level dynamics requires a structured validation framework.

## Validation Framework: Mapping Epigenetic Memory

Validating the architecture of the Epigenetic Detent requires clinical trials to transition from simply tracking weight loss to evaluating tissue-level biological adaptations after antiobesity therapy cessation. Building on progress made in mapping cellular changes through transcriptional profiling [20], we propose a translational framework pairing structured environmental modification with longitudinal tissue profiling to define an adipose multiomic signature (AMOS).

Participants undergoing structured withdrawal from glucagon-like peptide-1 (GLP-1) and glucose-dependent insulinotropic polypeptide (GIP) receptor agonist therapy could be evaluated through serial - abdominal subcutaneous white adipose tissue biopsies. To bypass the tissue-shearing limitations of traditional fine-needle aspirations and the greater procedural morbidity of open surgical biopsies, tissue architecture can be captured using a modified 6-mm Bergström side-cutting needle paired with syringe-assisted vacuum. This minimally invasive technique yields ∼1.5 g of unfragmented adipose cores per sampling, ensuring complete representation of the stromal vascular fraction and extracellular matrix required for single-cell transcriptomic and epigenomic sequencing. This approach aims to determine whether the adipocyte epigenetic marks identified in human fat tissue [3] are erased or maintained following antiobesity therapy withdrawal. By tracking chromatin accessibility profiles (via assay for transposase-accessible chromatin with high-throughput sequencing) and transcriptional programs, such as *PPARG* and *SREBF1* signaling alongside localized inflammatory networks, this tissue biopsy model can isolate peripheral adipocyte memory from central neuroendocrine drivers. Although central hypothalamic structures cannot be directly sampled, tracking the longitudinal behavior of these adipose tissue marks provides an accessible, tissue-level proxy for whether the body has achieved a true reset of its defended high-adiposity set-point.

Because extracting AMOS signatures requires transcriptomic data from invasive biopsies, peripheral blood mononuclear cell methylation profiles should be cross-validated against these adipose tissue marks to develop a blood-based analog: the blood-based AMOS (blAMOS). Validating this blood-based proxy will allow clinicians to track a patient’s true metabolic recovery without requiring repeated tissue biopsies. Ultimately, this approach provides a practical tool to determine whether a patient has achieved true biological remission compared with an Incretin Mirage, helping clinicians safely guide the transition away from long-term drug dependence toward sustained lifestyle and environmental maintenance.

## Conclusions and Future Directions

The rapid weight regain observed after discontinuing incretin-based therapies reveals that these pharmacotherapies, while effective, reduce body weight without resolving the underlying cellular biology. If the Incretin Mirage accurately describes this dissociation between a lower body weight and a persistent epigenetic memory, then GLP-1 and GIP receptor agonists should not be viewed as standalone cures for chronic obesity. Instead, they represent a critical, time-limited clinical window of opportunity. During this window, apparent weight maintenance masks underlying epigenetic marks—the molecular mechanism of the Epigenetic Detent that may continue to defend the prior high-adiposity set-point.

Achieving a complete cellular metabolic reset requires using this therapeutic window to disengage the Detent through combined pharmacotherapy, lifestyle modification, and environmental restructuring. Future obesity management must shift its clinical endpoint from short-term weight loss to long-term metabolic stabilization, ensuring that clinical care ultimately prioritizes the cell over the scale. Recognizing obesity as a modifiable, stage-dependent biological process has implications for precision medicine (see Outstanding Questions).

## Outstanding Questions

• What molecular checkpoints determine whether adult human adipocytes retain the “Epigenetic Detent” or undergo a complete cellular metabolic reset after incretin therapy?
• How can clinical investigators differentiate a temporary, drug-dependent reduction in adipose cell volume from a durable recalibration of the defended high-adiposity set-point?
• Does incretin therapy initiation during adolescent developmental windows prevent the establishment of obesity-associated DNA methylation patterns, or merely delay their phenotypic expression?
• Can the AMOS, derived from tissue biopsies, predict postdiscontinuation weight trajectories before treatment cessation?
• Can the blAMOS proxy accurately reflect the chromatin accessibility changes occurring in subcutaneous adipose tissue across diverse clinical populations?
• What targeted epigenetic-editing strategies in preclinical models, or environmental restructuring approaches in clinical settings, can be paired with time-limited incretin therapies to disengage the “Epigenetic Detent”?

## Author contributions

The authors’ responsibilities were as follows – RK: conceptualized the perspective, conducted the literature review, and drafted the initial manuscript; GHA: provided critical oversight and revised the manuscript for scientific rigor; and both authors: contributed to the refinement of the final draft and approved the final manuscript.

## Declaration of generative AI and AI-assisted technologies in the writing process

During the preparation of this work, the authors used Google Gemini for language editing and grammatical refinement. The authors reviewed and edited the output as needed and take full responsibility for the content of the published article.

## Funding

This research received no specific grant from any funding agency in the public, commercial, or non-profit sectors. The conceptual ideas were developed by building on prior research supported by the Canadian Institutes of Health Research (CIHR) Institute of Nutrition, Metabolism and Diabetes.

## Conflict of interest

The authors report no conflicts of interest.
